# Fast machine learning image reconstruction of radially undersampled k-space data for low-latency real-time MRI

**DOI:** 10.1371/journal.pone.0334604

**Published:** 2025-11-17

**Authors:** Johanna Topalis, Jakob Dexl, Katharina Jeblick, Rabea Klaar, Christopher Kurz, Timo Löhr, Andreas Mittermeier, Balthasar Schachtner, Anna Theresa Stüber, Tobias Weber, Philipp Wesp, Jens Ricke, Max Seidensticker, Guillaume Landry, Michael Ingrisch, Olaf Dietrich

**Affiliations:** 1 Department of Radiology, LMU University Hospital, LMU Munich, Munich, Germany; 2 relAI – Konrad Zuse School of Excellence in Reliable AI, Garching, Germany; 3 Munich Center for Machine Learning (MCML), Munich, Germany; 4 Comprehensive Pneumology Center (CPC-M), Member of the German Center for Lung Research (DZL), Munich, Germany; 5 Department of Radiation Oncology, LMU University Hospital, LMU Munich, Munich, Germany; 6 Department of Informatics, LMU Munich, Munich, Germany; 7 Department of Statistics, LMU Munich, Munich, Germany; 8 Bavarian Cancer Research Center (BZKF), Munich, Germany; 9 German Cancer Consortium (DKTK), Partner Site Munich, A Partnership Between DKFZ and LMU University Hospital Munich, Munich, Germany; Kyung Hee University, KOREA, REPUBLIC OF

## Abstract

Fast data acquisition and fast image reconstruction are essential to enable low-latency real-time magnetic resonance (MR) imaging applications with high temporal resolution such as interstitial percutaneous needle interventions or MR-guided radiotherapy.

To accelerate the image reconstruction of radially undersampled 2D k-space data, we propose a machine learning (ML) model that consists of a single fully connected linear layer to interpolate radial k-space data to a Cartesian grid, followed by a conventional 2D inverse fast Fourier transform. This k-space-to-image ML model was trained on synthetic data from natural images. It was evaluated with respect to image quality (mean squared error (MSE) compared to ground truth where available) and reconstruction time both on synthetic data with undersampling factors *R* between 2 and 10 as well as on radial k-space data from MR measurements on two different MRI systems. For comparison, conventional non-iterative zero-filling non-uniform fast Fourier transform (NUFFT) reconstruction and compressed sensing (CS) reconstruction were used.

On synthetic data, the ML model achieved better median MSE values than the non-iterative NUFFT reconstruction. The interquartile ranges of the MSE distributions overlapped for the ML and CS reconstructions for all *R*. Reconstruction times of the ML approach were shorter than for NUFFT and substantially shorter than for CS reconstructions. The generalizability (for real MRI data) of the ML model was demonstrated by reconstructing 0.35-tesla MR-Linac dynamic measurements of three volunteers and phantom data from a diagnostic 1.5-tesla MRI system; the median reconstruction time for the coil-combined images was much shorter than for the conventional approach (ML: <4ms; NUFFT: ≈60−90ms).

The proposed ML model reconstructs MR data with reduced streaking artifacts compared to non-iterative NUFFT techniques and with extremely short reconstruction times; thus, it is ideally suited for rapid low-latency real-time MR applications.

## Introduction

Magnetic resonance imaging (MRI) provides excellent soft-tissue contrast with complete anatomic coverage without exposing the patient to ionizing radiation. These distinctive properties make MRI extremely suitable not only for diagnostic imaging, but also for interventional applications such as image-guided interstitial percutaneous needle interventions (e. g., liver biopsies or tumor ablations) [1–10] or MRI-guided radiotherapy (MRgRT) with MR-Linacs [11–18]. While for diagnostic MRI, the image quality is of the highest importance, in these interventional procedures, the need for low-latency real-time imaging with best possible temporal resolution is particularly pronounced. To meet this need, two essential parts of the overall imaging process must be optimized with respect to speed: data acquisition, which involves collecting raw k-space data, and image reconstruction, i. e., processing of these acquired data to produce the final images [19].

To improve temporal resolution by reducing the acquisition time of each single image, k-space data can be acquired with highly undersampled non-Cartesian trajectories such as spiral [20] or radial [21–23] trajectories. The repeated sampling of the k-space center at different times makes these approaches particularly robust against motion, and, hence, suitable for real-time MR applications in the thorax or abdomen. However, using non-Cartesian (e. g., radial) trajectories comes with several drawbacks during image reconstruction. Undersampled data points on non-Cartesian trajectories need to be interpolated (“regridded”) to a Cartesian grid by non-uniform fast Fourier transform (NUFFT) algorithms [24], which, however, may result in substantial (streaking) artifacts due to missing data in k-space. To mitigate these artifacts, iterative methods such as the compressed sensing (CS) [25] approach have been developed, which, however, have much longer processing times [26]. This makes both of these conventional approaches insufficient for reconstruction tasks where both a fast reconstruction of images and sufficient image quality are required.

Machine learning (ML) has proven its potential for reconstructing undersampled k-space data [27,28]. ML models have been employed, e. g., for k-space-to-image domain [29–34] learning. For supervised training, undersampled k-space data and the corresponding ground truth (i. e., the image) are provided to the ML model to find a suitable transformation between the input and output. During inference, only the undersampled k-space is provided to the model, which then applies the learned transformations to reconstruct an image [27]. A notable example of this approach is the work by Waddington *et al*. [35] who addressed the need for a fast reconstruction of radial k-space data in the context of MRgRT and demonstrated the ability of a k-space-to-image neural network to reconstruct radial MR data with high temporal resolution. However, one limitation of the applied model named AUTOMAP (originally proposed by Zhu *et al*. [34]) is its relatively high number of model parameters [34,36,37]. The large size of the model and the large number of computations required even for inference make its application in clinical practice potentially difficult and less time-efficient than employing smaller models with fewer parameters. Furthermore, as discussed by Wang *et al*. [37], instead of relying on the fast Fourier transform (FFT), which is a well-established mathematical operation that provides highly efficient k-space-to-image domain conversion, AUTOMAP has to learn this transformation during training.

To address these challenges, we propose an ML model with a shallow, but fully connected architecture that enables a highly accelerated reconstruction of undersampled radial k-space data when compared to conventional approaches. In contrast to previous models such as AUTOMAP, our approach employs an explicit FFT, which simplifies the learning process and reduces model complexity. The reduced memory and computational requirements of this model make it more suitable for low-latency real-time applications. Moreover, by only training the model with synthetic data, we enable fast adaptation to varying undersampling factors without requiring new training data collection on the MRI scanner.

The purpose of this study is to develop and evaluate a novel machine learning model that can rapidly reconstruct MR images from highly undersampled radial k-space data, thereby enabling fast low-latency real-time MR imaging applications with improved temporal resolution and reduced artifacts.

## Materials and methods

This section first introduces the newly proposed ML model for image reconstruction, including its architecture, training process, and inference methodology. Next, we introduce the conventional reconstruction methods used for comparison in this study. The final subsections describe the evaluation of the proposed model on synthetic data and, to assess the model generalizability, on real-world MRI data, including phantom data and human in-vivo data.

### Machine learning reconstruction

#### Machine learning model.

**Fig 1:**
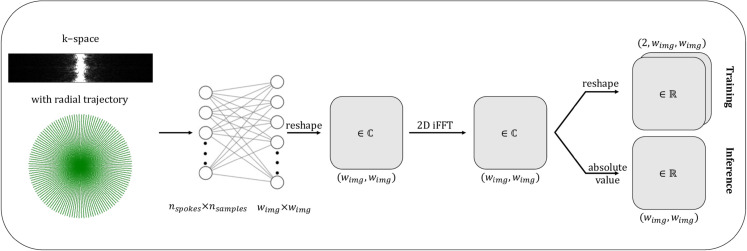
Architecture of the machine learning model. The real and imaginary part of the complex-valued radial k-space are stacked in the batch dimension before being passed to the machine learning model. The model consists of a linear layer (without bias) with $n_{\text{spokes}}\times n_{\text{samples}}$ input nodes and $w_{\text{img}}\times w_{\text{img}}$ output nodes. The output is then reshaped to a complex-valued quadratic matrix. By applying a 2D iFFT a complex-valued image is obtained. While the complex-valued image is split in its real and imaginary part for calculating the loss during training, the magnitude image is calculated during inference.

Our ML model – further referred to as ML model – was implemented in the PyTorch framework [38] (version 2.0.0). We utilize a single fully connected layer with trainable weights A and without a bias component or activation function, i. e., a purely linear model ($\text{output}=\text{input}\;\times \;A^{T}$). The input data are supplied in a flattened $n_{\text{spokes}}\times n_{\text{samples}}$-dimensional vector representation. Since the applied fully connected network operates only on real-valued data and parameters, the real and imaginary parts of the radial k-space data were handled separately and identically. By stacking the vectorized representations of the real and imaginary parts in the batch dimension, weight-sharing concepts were incorporated to simultaneously transform both parts using a single network. The output dimension corresponds to the desired spatial resolution of $w_{\text{img}}\;\times \;w_{\text{img}}$, which is restored through reshaping. After collecting both predictions (real and imaginary), a Cartesian 2D inverse fast Fourier transform (iFFT) transforms the estimated k-space data to image space to obtain a complex-valued image. The model architecture is summarized in Fig 1.

#### Synthetic training data.

We constructed a synthetic dataset with 200,000 samples (i. e., complex-valued images and corresponding radial k-space data) for training, 50,000 samples for validation, and 1,000 samples for testing from the ImageNet Large Scale Visual Recognition Challenge 2012 (ILSVRC2012) image classification and localization dataset [39,40]. First, ImageNet images were transformed to grayscale images, rescaled to the defined image size $\left(w_{img},w_{img}\right)$, and each image was min-max normalized to intensities between 0 and 1. Phase information for each image was generated based on a low-contrast high-pass filtered magnitude image (min-max normalized between –0.25 and 0.25), to which spatially slowly varying random phase data were added (min-max normalized between −π and *π*). Combining the magnitude, *M*, and phase information, *P*, as Mexp(iP), yielded an MR-like complex-valued image.

Radial k-space data were defined to lie on trajectories $k_{x,y}(\phi ,m)$ with $n_{\text{spokes}}$ spokes (with spoke angle *ϕ*) and $n_{\text{samples}}$ samples per spoke (with sample index *m*):

$$(\begin{array}{c} k_{x}(\phi ,m)\\ k_{y}(\phi ,m) \end{array})=(\begin{array}{c} \frac{2\pi m}{n_{\text{samples}}}\cos(\phi )\\ \frac{2\pi m}{n_{\text{samples}}}\sin(\phi ) \end{array}),$$ (1)

with $-n_{\text{samples}}/2+1\leq m\leq n_{\text{samples}}/2$.

To obtain (complex-valued) radial k-space data from the complex-valued images, a forward NUFFT with a radial k-space trajectory was performed using the PyNUFFT package [41] (version 2023.2.1) with a square image size of $\left(w_{\text{img}},w_{\text{img}}\right)$, k-space size of $\left(w_{\text{ksp}},w_{\text{ksp}}\right)$, and an interpolation kernel size of (6,6). For training, validation, and testing of the ML model, all k-space data were divided by the same normalization factor of $w_{\text{img}}^{2}$.

#### Machine learning training.

The ML reconstruction model was trained end-to-end with 200,000 samples from the training set and continuously validated with 50,000 samples from the validation set. The network was trained using the Adam [42] optimizer with a learning rate of $5\times 10^{-6}$ (with a reduce-on-plateau learning-rate scheduler with a patience of 5, a reduction factor of 0.8, $\epsilon =10^{-10}$), $\beta_{1}=0.9$, $\beta_{2}=0.98$, $\epsilon =10^{-9}$, and the mean squared error (MSE) loss function (evaluated on the real and imaginary image parts) to account for reconstruction quality.

A batch size of 128 complex-valued samples was selected for all undersampling factors. Training was stopped if the best loss did not improve by more than δ=0.0001×best_loss for 30 epochs. To improve the robustness of the network, we introduced spoke dropout during training. For this, the k-space values of $\frac{n_{\text{spokes}}}{8}$ randomly selected spokes (different in each epoch) were set to zero for every input. Additionally, uniformly distributed random noise between 0.8 and 1.2 was multiplied to the input data during training.

#### Machine learning inference.

During inference the absolute value of the complex-valued prediction is calculated after performing the 2D iFFT (Fig 1). When reconstructing k-space measurements acquired with multiple coils, all coil k-space datasets were simultaneously passed through the model as a batch to accelerate the reconstruction time. To yield one coil-combined magnitude image, these magnitude coil images were combined with a root-sum-of-squares algorithm. To make the CPU reconstruction more competitive (e. g., for cases where no GPU is available), oneDNN Graph [43] was used to accelerate the inference on the CPU.

### Conventional reconstruction

For comparison purposes, we reconstructed the radial k-space data with established conventional approaches from the Berkeley advanced reconstruction toolbox (BART) [44,45] (version v0.7.00). As a non-iterative approach, the BART adjoint NUFFT algorithm with zero-filling (bart nufft -a ...) was applied. A density correction function $\sqrt[{4}]{|k{|}^{4}+D^{4}}\approx |k|$ with $|k|=\sqrt{k_{x}^{2}+k_{y}^{2}}$ was used to scale the radial k-space values before applying the adjoint NUFFT reconstruction. An empirically determined constant *D* (e. g., D≈0.0043 for *w*_*img*_ = 128 and $n_{\text{samples}}=256$) ensured that k-space values in the center were not scaled to 0.

For CS, we selected the BART parallel imaging and compressed sensing (PICS) tool with l1-wavelet regularization, utilizing a regularization $\lambda =10^{-5}$ and 2000 (maximum) iterations. The stepsize was scaled based on the maximum eigenvalue (bart pics -S -l1 -r 1e-5 -i 2000 -e ...). A constant sensitivity map of the image size $\left(w_{\text{img}},w_{\text{img}}\right)$ was selected for the reconstruction with PICS.

Lastly, the magnitude of the reconstructed image was calculated.

### Evaluation on synthetic data

To assess the general reconstruction performance of the ML model, we first conducted experiments with synthetic data, for which ground-truth images were available, allowing the calculation of image quality metrics on a large dataset.

For the experiments on the synthetic dataset, we selected a uniform trajectory as described in Eq 1 with spoke angles $\phi_{k}=\frac{k}{n_{\text{spokes}}}2\pi$ with $0\leq k\leq n_{\text{spokes}}\;-\;1$. For the desired image size of (128,128), a fully sampled radial trajectory requires $128\frac{\pi }{2}\approx 201$ spokes [46]. In this study, we assess the reconstruction performance for six different undersampling factors R={2,3,4,5,6,10} with $n_{\text{spokes}}=\{101,67,51,41,33,21\}$ and $n_{\text{samples}}=256$. For each trajectory, synthetic data for training, validation and testing were generated and ML models were trained.

We evaluated the reconstruction performance of our proposed ML reconstruction model by comparing the reconstruction quality for 1,000 synthetic samples in the test set to the performance of the conventional approaches.

Since real-world MR k-space data always contain noise, we additionally analyzed the behavior of the different reconstruction approaches with synthetic k-space data to which complex Gaussian noise was added. To generate such a test dataset with noise, we applied random (different for the real and imaginary part) normally distributed noise with a standard deviation of $\sqrt{2}\times \frac{1}{50}\times w_{\text{img}}$ to the radial k-space data of the noise-free synthetic test set. This noise level corresponds to a peak-signal-to-noise ratio of 50 in the fully sampled Cartesian case.

The ML CPU- and GPU-based reconstruction times were compared to the CPU NUFFT reconstruction time (no GPU-based adjoint NUFFT was implemented in the used version of BART) and the CPU- and GPU-based CS reconstruction times. Reconstruction times of all approaches were evaluated in an end-to-end fashion, including the time for loading the k-space data (saved in RAM), pre-processing steps (applying the density correction for the NUFFT reconstruction), the reconstruction, and post-processing (calculating the magnitude image; normalization of the NUFFT reconstruction).

### Evaluation in real-world applications

To demonstrate the generalizability of the proposed ML model, we also evaluated the reconstruction performance for phantom data acquired at a 1.5 T MRI system as used in MR-guided interventions and for in vivo data acquired at a 0.35 T MR-Linac as used in MRgRT. The ML MRI reconstructions were qualitatively compared to the conventional NUFFT reconstructions of the undersampled radial k-space data – which still achieve acceptable reconstruction times as observed in the experiments with synthetic data. We have refrained from a comparison with the CS approach because the reconstruction times were too long for the intended applications as already observed in the experiments with synthetic data.

#### MRI phantom data.

Radial k-space data of a static 3D abdominal phantom (triple-modality 3D abdominal phantom, model 057A, Computerized Imaging Reference Systems Inc., Norfolk, USA) were acquired with the same undersampling factors, *R*, and identical trajectories as for the synthetic data. These experiments were performed on a 1.5 T whole-body MRI system (MAGNETOM SolaFit, Siemens Healthineers, Erlangen, Germany) with a maximum gradient strength of 45 mT/m and slew rate of 200 T/m/s. 16 coil elements in the patient table and one interventional (single-channel) receive coil positioned on top of the phantom were used. Data of 2D slices with a slice thickness of 10 mm and a field of view of $384\;\times \;384\;{\text{mm}}^{2}$ were acquired sequentially in three perpendicular orientations with a spoiled gradient-echo sequence similar to the one we use for real-time needle guidance in liver interventions [5,8]. The sequence parameters were: echo time $T_{E}=3.42\;ms$, repetition time $T_{R}=7.31\;ms$, flip angle of $30^{\circ }$, and a bandwidth of 250 Hz/pixel. The resulting acquisition times per image are summarized in S3 Table. The ML reconstructions were performed with the same models that were also used for the experiments on the synthetic data.

#### In-vivo MR-Linac data.

Ethical approval for volunteer measurements (project number 21-0019) was granted by the local ethics committee (LMU Klinikum) and written informed consent was signed by all participants. The recruitment started at 11-Oct-2022 and is currently still ongoing.

In vivo radial k-space data of three healthy volunteers were acquired at a 0.35 T MR-Linac (MRIdian, ViewRay Inc., Cleveland, Ohio) with a maximum gradient strength of 18 mT/m and a slew rate of 200 T/m/s. Two 6-channel torso coils of the vendor were used to receive the MR signal. Data of 2D slices with a slice thickness of 10 mm and a field of view of $400\;\times \;400\;{\text{mm}}^{2}$ were acquired in coronal and sagittal orientations with a balanced steady-state free-precession sequence. The sequence parameters were echo time $T_{E}=1.28\;ms$, repetition time $T_{R}=2.55\;ms$, flip angle of 130^°^, and a bandwidth of 875 Hz/pixel. The sequence used a radial trajectory (Eq 1) with a non-uniform angle distribution with $\phi_{k}=\arctan(2\;\tan(\frac{k}{n_{\text{spokes}}}2\pi ))$. Four frames of radial data with 34 spokes (each subsampled from 136 denser spokes) were acquired sequentially over 500 repetitions. The acquisition time for one image was approximately 90 ms.

Four ML models – one for each shifted group of angles – were trained with synthetic data generated for $w_{\text{img}}=112$, $w_{\text{ksp}}=224$, $n_{\text{samples}}=224$, $n_{\text{spokes}}=34$. The median reconstruction time for the MR measurements was calculated over 500 repetitions in a similar end-to-end fashion as for the synthetic dataset. The measured time did not include the time for loading and preparing the raw data (e. g., saving it in a correct format for the NUFFT or ML reconstruction) but included the time for root-sum-of-squares combination of the coil reconstructions.

### Statistical evaluation

For the evaluations on synthetic data, image quality was quantified using the MSE and the structural similarity index measure (SSIM) [47] comparing the reconstructed image and the corresponding ground-truth image. For MSE and SSIM, we report median values as well as lower and upper quartiles for all undersampling factors. We refrained from statistical testing, as the large number of synthetic samples (n=1,000) would render even marginal differences statistically significant, without necessarily indicating meaningful or practically relevant effects [48,49]. Instead, we focused on descriptive statistics and the visual inspection of interquartile ranges (displayed in boxplots) and their overlaps to compare image quality across methods.

Since no ground-truth images were available for the measured radial MRI datasets, we analyzed the image quality visually. To assess reconstruction times – for synthetic data as well as MR measurements – we report median and interquartile values.

All pre-processing, training and evaluation steps were performed on an NVIDIA GeForce RTX 3090 system with one graphics processing unit (GPU) with 24 GB memory capacity, 10496 CUDA cores, and an AMD Ryzen 9 3950X 16-core/32-thread central processing unit (CPU) with 128 GB RAM.

## Results

### Evaluation on synthetic data

Training of the ML model with $n_{\text{spokes}}\times n_{\text{samples}}\times 128^{2}$ trainable weights (e. g., 423,624,704 for *R* = 2 and 88,080,384 for *R* = 10) required (dependent on the undersampling factors) between approximately 4.7 (for *R* = 10) and 22.1 hours (for *R* = 2).

**Fig 2:**
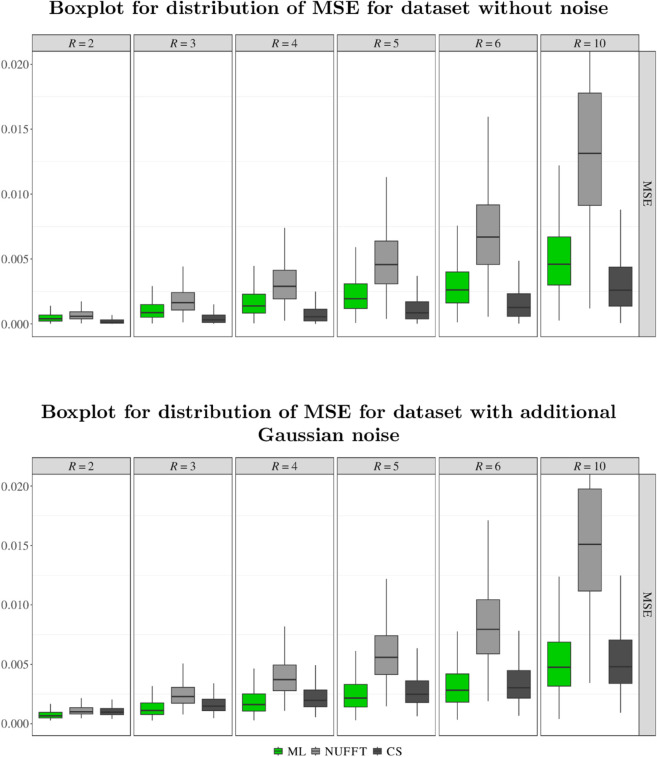
Distribution of mean squared error (MSE) values calculated for the reconstructions of synthetic test data without (top) and with (bottom) additional Gaussian noise for the varying undersampling factors, *R.* The boxes span over the range between the 25th and 75th percentile (IQR). The whiskers extend the IQR box by 1.5 times the IQR (or to the max/min values). The bold lines indicate the median values. Outliers are not displayed for better readability. ML = machine learning, NUFFT = non-uniform fast Fourier transform, CS = compressed sensing.

S1–S3 Figs display the reconstructions of example synthetic images from simulated k-space data with and without additional noise. Image quality evaluations for the test set are summarized in Fig 2 for MSE and in S4 Fig for SSIM. Generally, increasing the undersampling factors, i. e., accelerating the data acquisition, led to a stepwise worsening of the median MSE and SSIM values – and of the visual image quality – for all reconstruction approaches.

For data from the test set without noise (Fig 2, upper part), the best median MSE values were obtained with CS, followed by ML and NUFFT for all undersampling factors. The MSE box plots show a partial overlap of the interquartile ranges for the ML and CS reconstructions for all undersampling factors, but only for R≤4 when comparing the ML and NUFFT reconstruction.

With noise added to the k-space test set data (Fig 2, lower part), the median MSE calculated for the CS reconstructions increased by a factor of up to approximately 7.5 (for *R* = 2: $1.3\;\times \;10^{-4}$ vs. $9.7\;\times \;10^{-4}$), whereas substantially less relative increase was observed for the ML and NUFFT reconstructions. This resulted in marginally better median MSE values with the ML approach than with CS for all undersampling factors. For the ML and CS reconstruction, the intervals between lower and upper quartiles of the MSE distributions overlapped substantially for all *R*. Furthermore, the MSE box plots show no overlap of the interquartile ranges when comparing ML and NUFFT reconstructions for all undersampling factors R≥4, i. e., clearly better MSE values were obtained for the ML approach than for the NUFFT reconstruction of noisy data.

**Fig 3:**
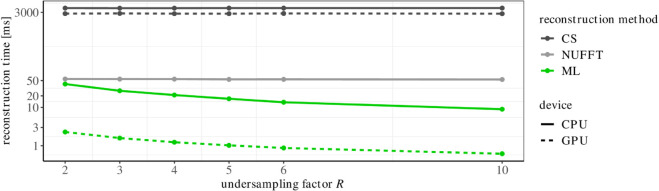
The median reconstruction times for the reconstructions of the synthetic data with a CPU (solid line) and GPU (dotted line) (on a logarithmic scale). ML = machine learning, NUFFT = non-uniform fast Fourier transform, CS = compressed sensing.

CPU reconstruction times were approximately 3800ms per image for CS and 55ms for NUFFT reconstruction (both independent of the undersampling factor). Much shorter CPU inference times were measured for the ML reconstruction, particularly at higher undersampling factors (Fig 3). For example, the ML median (lower quartile, upper quartile) CPU reconstruction time was 41(40,41)ms for *R* = 2 and decreased to 9(9,9)ms for *R* = 10. By running the ML reconstruction on a GPU, the processing times were strongly reduced to approximately 2(2,2)ms for *R* = 2 and even lower for higher undersampling rates. The GPU CS reconstruction time was reduced by approximately 1000ms compared to its CPU reconstruction.

### Evaluation in real-world applications

#### MRI phantom data.

**Fig 4:**
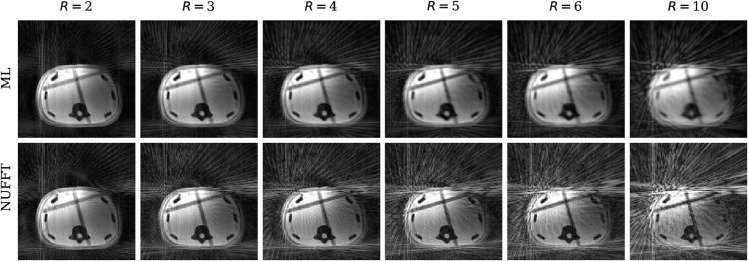
Reconstructions of undersampled k-space phantom measurements for varying undersampling factors, R. All images were windowed between 0 and the 99th percentile of the image intensity. ML = machine learning, NUFFT = non-uniform fast Fourier transform.

Fig 4 shows the coil-combined reconstructions of the acquired phantom data with the proposed ML approach and NUFFT for increasing undersampling factors 2≤R≤10. For *R* = 2, both compared reconstruction approaches resulted in acceptable image quality. For R≥3, increasingly stronger streaking artifacts worsen the image quality of the NUFFT reconstructions, while acceptable image quality was achieved for the ML approach for undersampling factors R≤6. For an undersampling factor of *R* = 10, both reconstruction approaches failed to reconstruct images with sufficient image quality.

#### In-vivo MR-Linac data.

**Fig 5:**
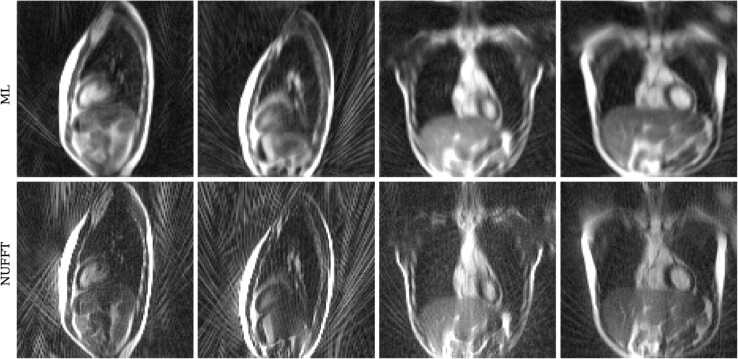
Exemplary reconstructions of MR-Linac in vivo measurements of healthy participants. All images were windowed between 0 and the 96th percentile (sagittal) or 98th percentile (coronal) of the image intensity. ML = machine learning, NUFFT = non-uniform fast Fourier transform. (A movie of the whole dynamic acquisitions is provided as supporting information.)

Fig 5 shows four coil-combined ML and NUFFT reconstructions of k-space data acquired in volunteers on an MR-Linac system. All reconstructed frames are presented as a movie in the supplementary files (S1 Movie). The ML reconstruction showed good image quality and much less intensity of streaking artifacts than the NUFFT reconstruction. The median CPU reconstruction times of the ML model were approximately 11ms, while the NUFFT approach reconstructed the coil-combined images within approximately 75ms. Utilizing a GPU for the calculations strongly accelerated the reconstruction with ML to about 1ms (Table 1).

**Table 1:** Median (interquartile range) reconstruction times for ML and NUFFT reconstruction for in-vivo MR-Linac data.

Method	Device	Reconstruction time [ms]
ML	CPU	10.7 (10.6, 10.9)
ML	GPU	1.0 (1.0, 1.0)
NUFFT	CPU	74.9 (72.9, 77.4)

## Discussion

In this study, we proposed an ML model for fast MR image reconstruction from undersampled radial k-space data and compared it to conventional reconstruction approaches. The model was motivated by applications that require fast low-latency real-time MR imaging such as interstitial percutaneous needle interventions or MRgRT. We demonstrated that the proposed ML model reconstructs MR data substantially faster and with reduced streaking artifacts compared to non-iterative NUFFT techniques despite being trained only on synthetic data. Slower iterative reconstruction techniques such as CS can result in better (for noise-free data) or comparable (for data with additional noise) image quality (MSE), but at much longer reconstruction times, that are not suitable for real-time MR applications.

The proposed k-space-to-image ML model consists of a linear operator with trained weights and a Cartesian 2D iFFT. The linear part is trained to interpolate radial k-space data to a Cartesian grid and to estimate missing k-space values in order to obtain an approximation of fully sampled Cartesian k-space data. The complex-valued image is then calculated by explicitly performing a Cartesian 2D iFFT. Since there is no uniform spatial relation between neighboring input data points (e. g., if radial k-space data are saved in the format $\left(n_{\text{spokes}},n_{\text{samples}}\right)$), we decided to use a fully connected model (similar to the first layer of the AUTOMAP model [34]), instead of a conventional convolutional neural network. By minimizing the depth of the model, a low inference time was ensured, which is a major requirement for low-latency MR imaging.

ML approaches have presented their potential to solve a variety of tasks by modeling a relationship between input and output data without the need for domain knowledge. However, incorporating domain knowledge into ML models can be advantageous for certain applications. For instance, it can help to simplify the training process or to overcome problems arising from insufficient training data and to consequently boost the performance [50]. Domain knowledge has also been successfully incorporated to ML models for various tasks in the medical domain such as deep-learning MR reconstruction [51]. We decided to incorporate domain knowledge to the proposed reconstruction model by explicitly performing a 2D iFFT to calculate a complex-valued image from the fully sampled Cartesian grid similar to earlier publications [29,52]. Even though Zhu *et al*. [34] have shown with their model named AUTOMAP that an ML model is capable of approximating the manifold between k-space and the image domain (and learning the Fourier transformation), the iFFT is a very fast and memory-efficient mathematical transformation which does not necessarily need to be approximated by a neural network (as discussed by Wang *et al*. [37]). By interpolating the radial k-space values to a Cartesian grid independently for real and imaginary parts, reducing the number of fully connected layers to one, calculating the 2D iFFT directly, and not using convolutional layers, we were also able to reduce the number of model parameters by a factor of >2.5 (considerably more for higher undersampling factors) in comparison to the AUTOMAP model (e. g., for a trajectory with 51 spokes and 256 samples: 213,909,504 vs. 696,394,497 model parameters, i. e., a reduction factor of 3.3). This consequently reduced the required memory as well as the inference time. With a similar motivation, the decomposed-AUTOMAP (dAUTOMAP) network [53] also addressed the non-scalability of AUTOMAP. However, for highly undersampled radial k-space data, dAUTOMAP has been demonstrated to reconstruct overly smoothed images [54].

To improve the robustness of our ML approach, we tried to guide the fully connected network to learn some desirable properties present in conventional regridding algorithms, which, e. g., can still provide sufficient results after various spokes are set to zero. Thus, we introduced spoke dropout, where the k-space values of a set of random spokes were set to zero during training.

For the training of ML models, sufficiently many and diverse data are required [55,56]. In the past, public [57,58] (e. g., the fastMRI dataset [59,60]) and non-public datasets [61] were used for developing ML-based models for MR image reconstruction or processing. To our knowledge, no public datasets with radial k-space data matching our interventional MR and MRgRT protocols are available. Zhu *et al*. [34] have presented good generalizability of their MR reconstruction model AUTOMAP to real k-space measurements after training it only with preprocessed natural images. Waddington *et al*. [35] have trained the AUTOMAP model with the YouTube-8M dataset [62] and have demonstrated that the model was able to learn motion properties from the video data which consequently improved the target tracking accuracy for dynamic MR data. Jaubert *et al*. [63] showed that training dynamic MR image reconstruction models on natural videos yields no significantly different results compared to training on true cardiac MR data. Following these promising results, our proposed ML model was trained solely on preprocessed natural images and the corresponding radial k-space data. We created synthetic images that have similar characteristics to MR images by combining a greyscale magnitude image with a phase image and calculated radial k-space data by performing a NUFFT. Such an approach not only eliminates the need for (huge amounts of) real-word training data, which is a big challenge when training an ML MR reconstruction model [28], but also allows an easy adaption of the training data, e. g., for other undersampling factors without the need of acquiring additional data at the MR scanner.

To allow an evaluation of the reconstruction approaches on a large dataset, where also ground truth data was available (which is generally not the case for MR measurements), the methods were first tested on the synthetic test dataset with 1,000 images and a uniform radial k-space trajectory. As expected, the iterative CS reconstructions showed better image quality (MSE and SSIM) than the images reconstructed with the proposed ML model for the synthetic test dataset without noise. However, the median MSE calculated for the ML model was more robust to additional Gaussian noise added to the k-space data than the CS approach. Even more important, the proposed ML reconstruction model achieved much faster inference times compared to the CS method.

Since data are missing in the k-space periphery due to undersampling, images reconstructed with the zero-filling NUFFT contained strong streaking artifacts leading to worse median MSE and SSIM values especially for higher undersampling factors. The ML model successfully reconstructed radial k-space data for undersampling factors up to *R* = 6 with fewer artifacts than in the NUFFT reconstruction, but with a slightly more blurred appearance. To make sure that the observed MSE and SSIM differences were not only caused by different levels of image smoothing, we performed a supplementary comparison in which we applied Gaussian filtering to the NUFFT-reconstructed data, which, however, did not improve MSE/SSIM to the level of the ML approach (S5 and S6 Figs). For instance, the images in S5 Fig illustrate similar levels of smoothing in ML reconstructions on the one hand and in NUFFT reconstructions with a Gaussian filter width σ=1.0px on the other hand; however, the amount of streaking artifacts is considerably higher in filtered NUFFT reconstructions. This is also confirmed in S6 Fig, which demonstrates best image quality within the filtered NUFFT reconstructions for σ=1.0px, but the resulting MSE and SSIM values are still worse than the corresponding values of the ML reconstructions. This demonstrates that the proposed ML model reduces artifacts and is (despite some image smoothing) clearly superior to the conventional NUFFT reconstruction with or without additional Gaussian filter.

All this indicates that even though the proposed linear model simplifies the regridding task to a simple matrix multiplication $\text{output}=\text{input}\;\times \;A^{T}$, where the parameters of the weight matrix A were learnt, satisfying image quality can be achieved in the reconstructions.

The CPU inference times of the ML model were superior for all undersampling factors. Deploying the ML reconstruction on a GPU reduced the reconstruction time to below 3ms, which is substantially shorter than reconstruction times of the conventional approaches. There are also other state-of-the-art CPU and GPU implementations of NUFFT such as gpuNUFFT [64], TorchKbNufft [65] or cuFINUFFT [66] which could potentially lead to faster processing times for NUFFT reconstructions as the here evaluated BART NUFFT implementation. However, since the non-iterative NUFFT approach is limited not by reconstruction time, but by image quality, we did not further investigate other NUFFT implementations.

Similar observations were made with respect to image quality and reconstruction time for the reconstruction of real MR acquisitions. The model, which was only trained on synthetic data, generalized well to real k-space data of a phantom acquired on a 1.5 T MRI system and of volunteers acquired on a 0.35 T MR-Linac, and achieved perceptually good image quality. The NUFFT reconstructions showed again much stronger streaking artifacts.

For both the ML approach and the NUFFT, the reconstruction times of multi-channel MRI phantom measurements were not relevantly (and not proportionally) higher than for a single image in the synthetic dataset. CPU- and GPU-based ML reconstruction times were substantially faster than the CPU-based NUFFT. The (GPU-based) ML reconstruction time below 3ms is sufficiently short to enable reconstruction with a high temporal resolution as required in real-time MR applications.

This study has limitations. The reported reconstruction time measurements showed slight variations, e. g., due to background processes, and were only measured on one computer. Reconstruction times of an implementation on actual MR scanner hardware can be expected to behave qualitatively similar but may deviate in detail from the reported results.

The present proof-of-concept study focuses on the viability of the proposed ML model for the reconstruction of radial k-space data. Since the fully connected network interpolates k-space data to a Cartesian grid and estimates missing values, we expect it (with appropriate modifications) to be also applicable to other non-Cartesian acquisition schemes such as undersampled spiral trajectories. Since the proposed model was trained on synthetic data, the model can easily be trained for other acquisition schemes. However, the performance for other acquisition schemes was not evaluated here and must be examined in future studies.

In the following, additional possible future research directions are discussed. In this study, we did not utilize all available information from dynamic real-time MR imaging data, such as the high data redundancy in subsequent frames. Exploiting these redundancies can lead to better image quality, which, then, can be invested into higher undersampling factors. A 2D+time approach is proposed, e. g., by Jaubert *et al*. [67], in which five consecutive images are passed to the model which reconstructs a denoised version of the latest image. Zufiria *et al*. [68] presented a feature-based convolutional neural network (FbCNN) for the reconstruction of radially undersampled data acquired in MR-guided neurosurgery. The final image was created by integrating the refined features into the (fully sampled) reference image that was acquired in advance. However, this approach may not be suitable for real-time MR applications in organs with relevant motion such as demonstrated in our volunteer measurements of heart, lungs, and liver.

ML has also been used for improving the perceptual image quality of low-quality reconstructions [69]. In this context, mostly convolutional neural networks have been applied for noise [70,71] or artifact reduction [72–76], and for super resolution [77,78]. In future research, such a model could be applied after the reconstruction with our ML reconstruction model to further improve the perceptual image quality, but this might also result in longer (yet possibly still acceptable) reconstruction times. Furthermore, the proposed model could be expanded to include multi-coil reconstruction instead of combining individually reconstructed coil-images with a root-sum-of-squares approach. This would then allow the exploitation of parallel imaging principles [36] to take advantage of spatial correlations across receiver coils and consequently improve image quality.

To summarize, this study proposes an ML model for the fast reconstruction of undersampled radial 2D k-space data as obtained during real-time MR applications such as MR-guided interventions or MRgRT. The proposed ML model reconstructs MR data with reduced streaking artifacts compared to non-iterative NUFFT techniques and with extremely short reconstruction times; thus, it is ideally suited for rapid low-latency real-time MR applications.

## Supporting information

S1 Table Image quality results for the synthetic test set; k-space data without and with additional noise. Median (lower quartile, upper quartile) of mean squared error (MSE), structural similarity index measure (SSIM) values calculated for the reconstructions of synthetic test data for the varying undersampling factors, *R* for k-space data without and with additional Gaussian noise.
(PDF)

S2 Table Reconstruction time results for the synthetic test set. Median (lower quartile, upper quartile) of CPU and GPU reconstruction times calculated for the reconstructions of synthetic test data for the varying undersampling factors, *R* for k-space data without additional Gaussian noise.
(PDF)

S1 Fig Reconstruction of example image. Reconstructions of undersampled k-space data without (top) and with (bottom) additional Gaussian noise for varying undersampling factors, *R*, of a synthetic data sample. The ground truth magnitude image is shown in the left upper corner.
(PDF)

S2 Fig Reconstruction of example image. Reconstructions of undersampled k-space data without (top) and with (bottom) additional Gaussian noise for varying undersampling factors, *R*, of a synthetic data sample. The ground truth magnitude image is shown in the left upper corner.
(PDF)

S3 Fig Reconstruction of example image. Reconstructions of undersampled k-space data without (top) and with (bottom) additional Gaussian noise for varying undersampling factors, *R*, of a synthetic data sample. The ground truth magnitude image is shown in the left upper corner.
(PDF)

S4 Fig Boxplot for distribution of SSIM. Distribution of structural similarity index measure (SSIM) values calculated for the reconstructions of synthetic test data without (top) and with (bottom) additional Gaussian noise for the varying undersampling factors, *R*.
(PDF)

S5 Fig Example images of ML reconstruction and NUFFT reconstruction with Gaussian filters with varying *σ* for *R* = 6. (PDF)

S6 Fig Distribution of mean squared error (MSE) (top) and structural similarity index measure (SSIM) (bottom) values calculated for the reconstructions of synthetic test data with the ML approach and NUFFT reconstruction with Gaussian filters with varying *σ* for *R* = 6. (PDF)

S3 TableRadial trajectories with different undersampling factors, *R*, corresponding number of spokes, $n_{\text{spokes}}$, and samples per spoke, $n_{\text{samples}}$; acquisition time of measurements with a *T*_1_-weighted gradient echo sequence.(PDF)

S4 Table Median (lower quartile, upper quartile) CPU and GPU reconstruction times (measured over 100 repetitions) for k-space phantom measurements with varying undersampling factors *R*. (PDF)

S7 Fig Reconstructions of undersampled k-space phantom measurements (top: paracoronal orientation, bottom: parasagittal orientation) for varying undersampling factors, *R*. (PDF)

S1 Movie Video of exemplary reconstructions of MR-Linac in vivo measurements of healthy participants. (MOV)
