# Supplementary material for: Fast machine learning image reconstruction of radially undersampled k-space data for low-latency real-time MRI
Source: PLoS One. 2025 Nov 17;20(11):e0334604. doi: 10.1371/journal.pone.0334604 (PMC12622841; doi:10.1371/journal.pone.0334604)
Supplement: S1 Table — Median (lower quartile, upper quartile) of mean squared error (MSE), structural similarity index measure (SSIM) values calculated for the reconstructions of synthetic test data for the varying undersampling factors, R for k-space data without and with additional Gaussian noise. (PDF) [file pone.0334604.s001.pdf]

S1 Table. Image quality results for the synthetic test set; k-space data without and with additional noise

		without additional Gaussian noise		with additional Gaussian noise	
R	Algorithm	MSE ($\times 10^{-4}$)	SSIM ($\times 10^{-2}$)	MSE ($\times 10^{-4}$)	SSIM ($\times 10^{-2}$)
2	ML	3.9 (2.2, 6.9)	95.9 (94.3, 97.2)	6.6 (4.9, 9.6)	90.4 (87.7, 92.1)
	NUFFT	5.9 (3.9, 9.3)	92.8 (90.6, 94.4)	10.1 (8.1, 13.5)	86.0 (81.4, 89.1)
	CS	1.3 (0.5, 3.1)	98.5 (97.3, 99.3)	9.7 (7.6, 12.8)	88.1 (84.3, 90.9)
3	ML	8.6 (5.1, 15.0)	90.8 (87.9, 93.3)	11.2 (7.7, 17.5)	85.8 (83.5, 88.0)
	NUFFT	16.4 (10.7, 24.2)	83.3 (79.7, 86.2)	22.8 (17.2, 30.6)	76.5 (70.8, 80.5)
	CS	3.1 (1.2, 6.8)	96.3 (93.6, 98.1)	14.8 (11.0, 20.6)	84.8 (81.4, 87.4)
4	ML	13.8 (8.3, 22.8)	85.9 (82.1, 89.4)	16.1 (10.7, 25.1)	81.9 (79.2, 84.3)
	NUFFT	29.0 (19.3, 41.3)	75.4 (70.9, 78.8)	37.1 (27.8, 49.4)	68.8 (63.0, 73.1)
	CS	5.5 (2.3, 11.4)	93.4 (89.4, 96.4)	19.7 (14.3, 28.3)	81.7 (79.2, 84.4)
5	ML	19.4 (11.9, 30.9)	81.2 (76.9, 85.5)	21.6 (14.1, 33.0)	78.2 (74.5, 81.2)
	NUFFT	45.6 (30.9, 63.8)	68.5 (63.9, 72.3)	55.9 (41.4, 74.0)	62.4 (56.5, 66.6)
	CS	8.5 (3.8, 17.0)	90.1 (85.0, 94.3)	24.9 (17.8, 36.2)	79.1 (76.0, 81.7)
6	ML	26.1 (16.1, 40.0)	76.3 (71.3, 81.4)	28.1 (18.1, 42.0)	73.9 (69.5, 77.6)
	NUFFT	66.9 (45.7, 91.6)	61.8 (57.2, 65.4)	79.5 (58.8, 104.4)	56.0 (50.3, 60.0)
	CS	12.6 (5.9, 23.2)	86.0 (79.8, 91.5)	30.3 (21.5, 44.8)	76.0 (72.2, 79.3)
10	ML	46.0 (29.9, 67.0)	65.3 (58.7, 72.3)	47.5 (31.6, 68.6)	63.6 (57.7, 69.7)
	NUFFT	131.4 (91.2, 177.8)	47.9 (44.0, 51.3)	151.0 (111.7, 197.7)	43.1 (38.6, 46.6)
	CS	26.0 (13.7, 43.8)	75.0 (67.2, 82.5)	47.9 (33.8, 70.5)	67.5 (61.5, 73.0)

Median (lower quartile, upper quartile) of mean squared error (MSE), structural similarity index measure (SSIM) values calculated for the reconstructions of synthetic test data for the varying undersampling factors, R for k-space data without and with additional Gaussian noise. ML = machine learning, NUFFT = non-uniform fast Fourier transform, CS = compressed sensing.
