# Supplementary material for: Fast machine learning image reconstruction of radially undersampled k-space data for low-latency real-time MRI
Source: PLoS One. 2025 Nov 17;20(11):e0334604. doi: 10.1371/journal.pone.0334604 (PMC12622841; doi:10.1371/journal.pone.0334604)
Supplement: S2 Table — Median (lower quartile, upper quartile) of CPU and GPU reconstruction times calculated for the reconstructions of synthetic test data for the varying undersampling factors, R for k-space data without additional Gaussian noise. (PDF) [file pone.0334604.s002.pdf]

S2 Table. Reconstruction time results for the synthetic test set

R	Algorithm	time (CPU) [ms]	time (GPU) [ms]
2	ML	41 (40, 41)	2 (2, 2)
	NUFFT	55 (54, 56)	-
	CS	3862 (3797, 3916)	2776 (2737, 2832)
3	ML	27 (27, 27)	2 (2, 2)
	NUFFT	55 (54, 56)	-
	CS	3846 (3786, 3901)	2811 (2742, 2835)
4	ML	21 (21, 22)	1 (1, 1)
	NUFFT	55 (53, 56)	-
	CS	3847 (3791, 3900)	2769 (2738, 2829)
5	ML	17 (17, 17)	1 (1, 1)
	NUFFT	54 (52, 56)	-
	CS	3865 (3806, 3916)	2767 (2736, 2831)
6	ML	14 (14, 14)	1 (1, 1)
	NUFFT	54 (52, 56)	-
	CS	3865 (3811, 3915)	2805 (2739, 2829)
10	ML	9 (9, 9)	1 (1, 1)
	NUFFT	53 (52, 55)	-
	CS	3871 (3802, 3924)	2761 (2734, 2827)

Median (lower quartile, upper quartile) of CPU and GPU reconstruction times calculated for the reconstructions of synthetic test data for the varying undersampling factors, R for k-space data without additional Gaussian noise. ML = machine learning, NUFFT = non-uniform fast Fourier transform, CS = compressed sensing.
