# Supplementary material for: Fast machine learning image reconstruction of radially undersampled k-space data for low-latency real-time MRI
Source: PLoS One. 2025 Nov 17;20(11):e0334604. doi: 10.1371/journal.pone.0334604 (PMC12622841; doi:10.1371/journal.pone.0334604)
Supplement: S2 Fig — Reconstructions of undersampled k-space data without (top) and with (bottom) additional Gaussian noise for varying undersampling factors, R, of a synthetic data sample. The ground truth magnitude image is shown in the left upper corner. (PDF) [file pone.0334604.s004.pdf]

Reconstruction of example image from dataset without additional Gaussian noise

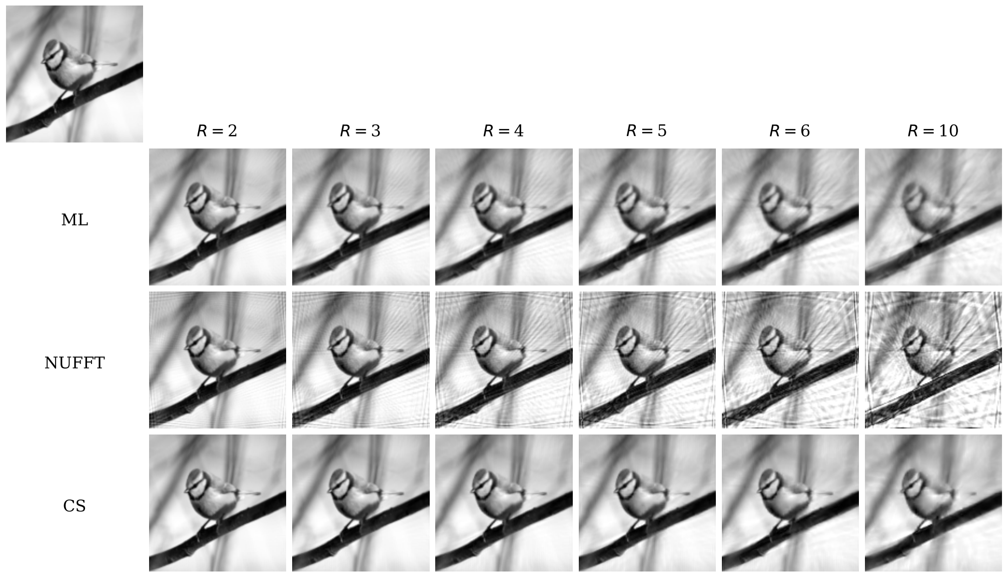

Reconstruction of example image from dataset with additional Gaussian noise

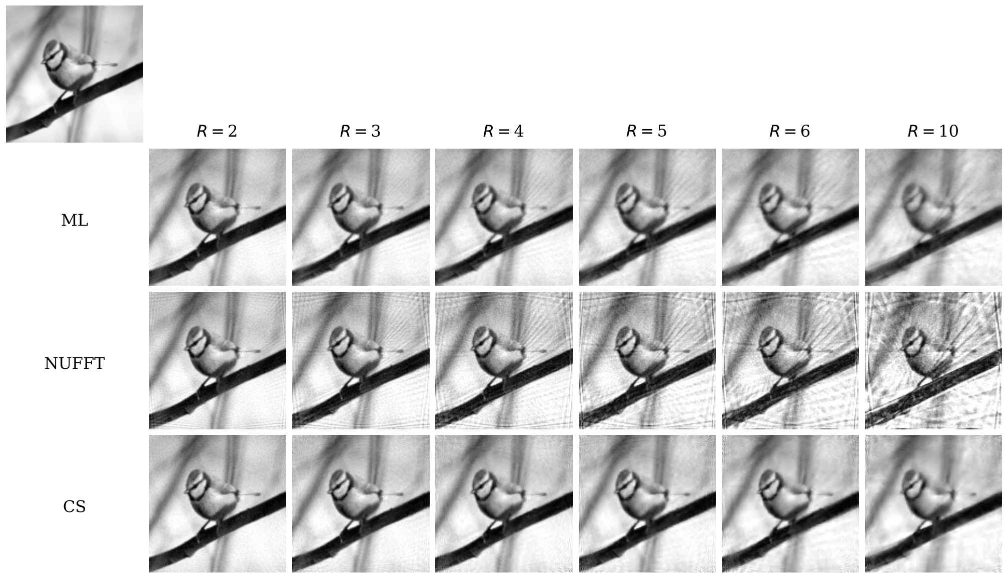

S2 Fig. Reconstructions of undersampled k-space data without (top) and with (bottom) additional Gaussian noise for varying undersampling factors, R, of a synthetic data sample. The ground truth magnitude image is shown in the left upper corner. ML = machine learning, NUFFT = non-uniform fast Fourier transform, CS = compressed sensing.
