# Supplementary material for: Fast machine learning image reconstruction of radially undersampled k-space data for low-latency real-time MRI
Source: PLoS One. 2025 Nov 17;20(11):e0334604. doi: 10.1371/journal.pone.0334604 (PMC12622841; doi:10.1371/journal.pone.0334604)
Supplement: S4 Fig — Distribution of structural similarity index measure (SSIM) values calculated for the reconstructions of synthetic test data without (top) and with (bottom) additional Gaussian noise for the varying undersampling factors, R. (PDF) [file pone.0334604.s006.pdf]

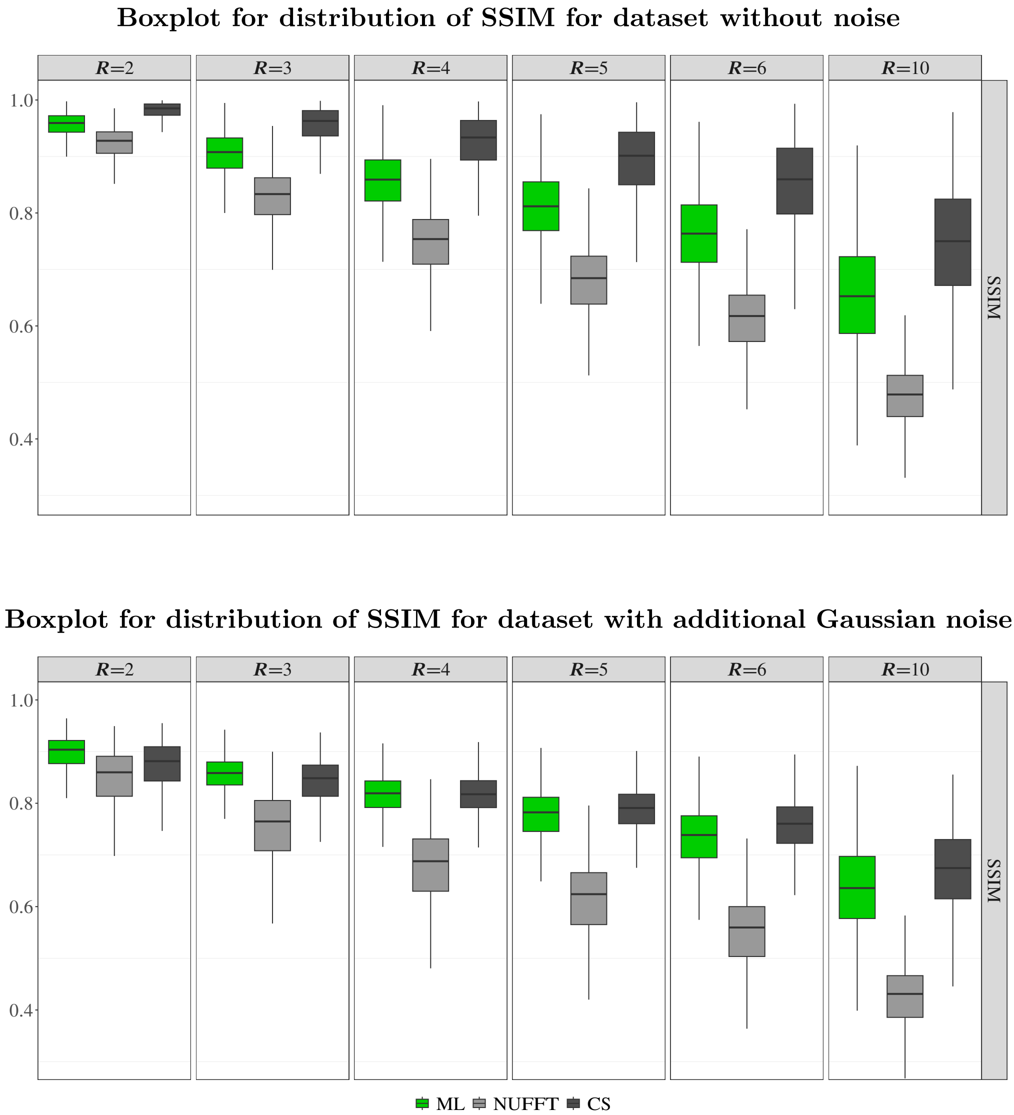

S4 Fig. Distribution of structural similarity index measure (SSIM) values calculated for the reconstructions of synthetic test data without (top) and with (bottom) additional Gaussian noise for the varying undersampling factors, R. The boxes span over the range between the 25th and 75th percentile (IQR). The whiskers extend the IQR box by 1.5 times the IQR (or to the max/min values). The bold lines indicate the median values. Outliers are not displayed for better readability. ML = machine learning, NUFFT = non-uniform fast Fourier transform, CS = compressed sensing.
