# Supplementary material for: Fast machine learning image reconstruction of radially undersampled k-space data for low-latency real-time MRI
Source: PLoS One. 2025 Nov 17;20(11):e0334604. doi: 10.1371/journal.pone.0334604 (PMC12622841; doi:10.1371/journal.pone.0334604)
Supplement: S5 Fig — (PDF) [file pone.0334604.s007.pdf]

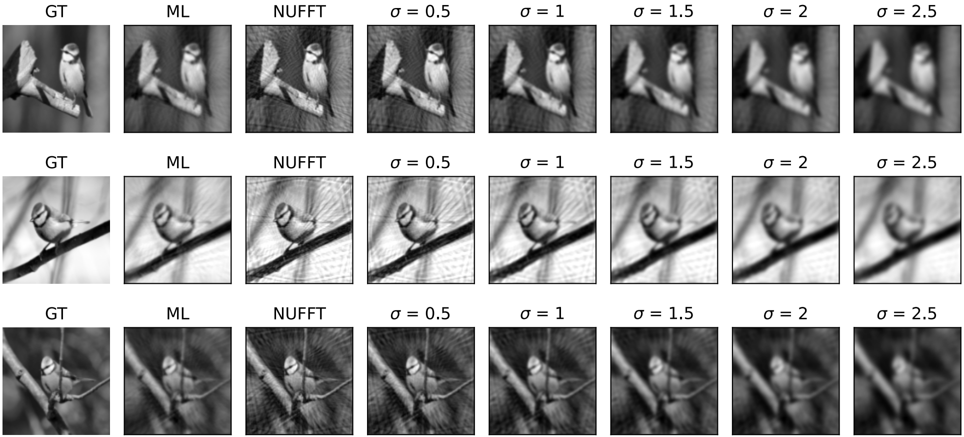

S5 Fig. Example images of ML reconstruction and NUFFT reconstruction with Gaussian filters with varying σ for $R = 6$. ML = machine learning, NUFFT = non-uniform fast Fourier transform.
