# Supplementary material for: Fast machine learning image reconstruction of radially undersampled k-space data for low-latency real-time MRI
Source: PLoS One. 2025 Nov 17;20(11):e0334604. doi: 10.1371/journal.pone.0334604 (PMC12622841; doi:10.1371/journal.pone.0334604)
Supplement: S6 Fig — (PDF) [file pone.0334604.s008.pdf]

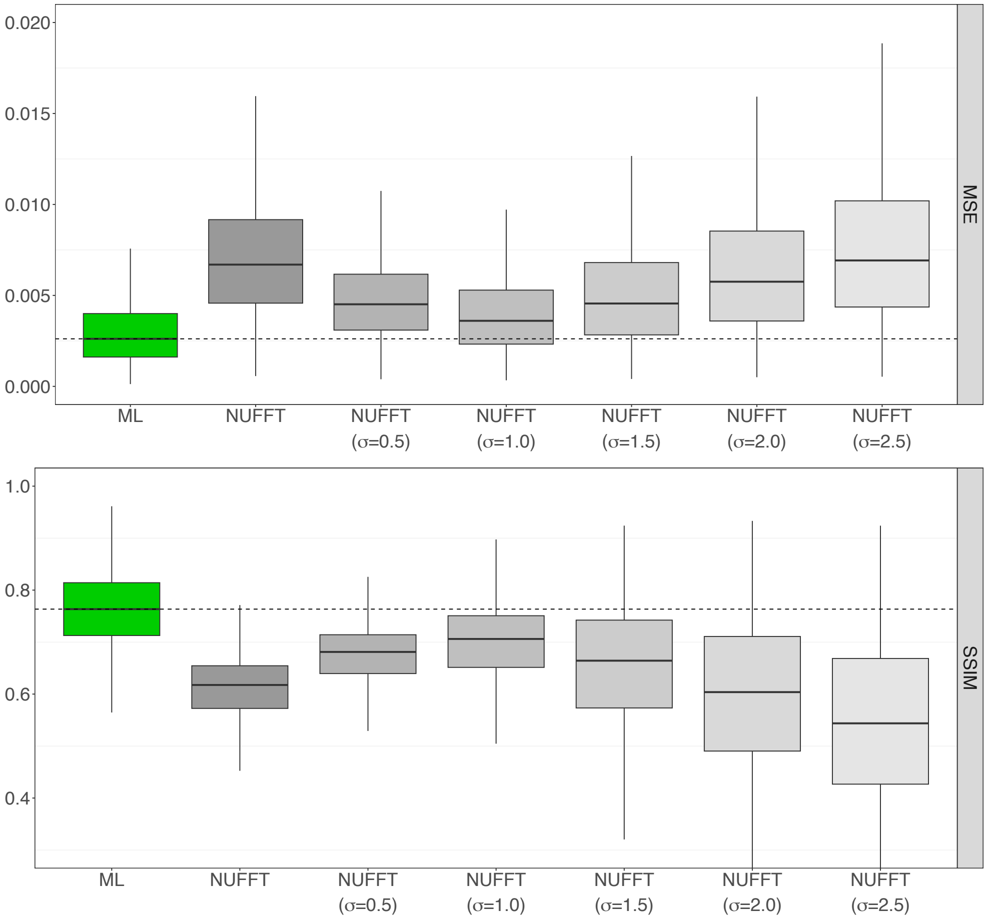

S6 Fig. Distribution of mean squared error (MSE) (top) and structural similarity index measure (SSIM) (bottom) values calculated for the reconstructions of synthetic test data with the ML approach and NUFFT reconstruction with Gaussian filters with varying σ for $R = 6$. ML = Machine learning, NUFFT = Non-uniform fast Fourier transform.
