# Supplementary material for: Fast machine learning image reconstruction of radially undersampled k-space data for low-latency real-time MRI
Source: PLoS One. 2025 Nov 17;20(11):e0334604. doi: 10.1371/journal.pone.0334604 (PMC12622841; doi:10.1371/journal.pone.0334604)
Supplement: S3 Table — (PDF) [file pone.0334604.s009.pdf]

S3 Table. Radial trajectories with different undersampling factors, R, corresponding number of spokes, n_{spokes}, and samples per spoke, n_{samples}; acquisition time of measurements with a T_1-weighted gradient echo sequence.

R	n_{spokes}	n_{samples} per spoke	acquisition time [ms]
2	101	256	738.31
3	67	256	489.77
4	51	256	372.81
5	41	256	299.71
6	33	256	241.23
10	21	256	153.51
