# Supplementary material for: Fast machine learning image reconstruction of radially undersampled k-space data for low-latency real-time MRI
Source: PLoS One. 2025 Nov 17;20(11):e0334604. doi: 10.1371/journal.pone.0334604 (PMC12622841; doi:10.1371/journal.pone.0334604)
Supplement: S4 Table — (PDF) [file pone.0334604.s010.pdf]

S4 Table. Median (lower quartile, upper quartile) CPU and GPU reconstruction times (measured over 100 repetitions) for k-space phantom measurements with varying undersampling factors R.

R	Algorithm	time (CPU) [ms]	time (GPU) [ms]
2	ML	55 (53, 57)	3 (3, 3)
	NUFFT	87 (85, 88)	-
3	ML	40 (38, 43)	2 (2, 2)
	NUFFT	78 (76, 80)	-
4	ML	30 (28, 31)	2 (2, 2)
	NUFFT	76 (75, 78)	-
5	ML	25 (23, 26)	2 (2, 2)
	NUFFT	73 (72, 75)	-
6	ML	21 (19, 22)	1 (1, 1)
	NUFFT	70 (68, 72)	-
10	ML	15 (13, 21)	1 (1, 1)
	NUFFT	66 (64, 69)	-

ML = machine learning, NUFFT = non-uniform fast Fourier transform.
